# 新辅助免疫治疗联合化疗在可手术的非小细胞肺癌的初步疗效评估

**DOI:** 10.3779/j.issn.1009-3419.2021.102.13

**Published:** 2021-06-20

**Authors:** 世杰 周, 学峰 郝, 大平 于, 树库 刘, 小庆 曹, 崇玉 苏, 小运 宋, 宁 肖, 云松 李, 威 杨, 丹 赵, 敬慧 王, 志东 刘, 绍发 许

**Affiliations:** 1 101149 北京，首都医科大学附属北京胸科医院胸外科 Department of Thoracic Surgery, Beijing Chest Hospital, Capital Medical University, Beijing 101149, China; 2 101149 北京，首都医科大学附属北京胸科医院病理科 Department of Pathology, Beijing Chest Hospital, Capital Medical University, Beijing 101149, China; 3 101149 北京，首都医科大学附属北京胸科医院肿瘤内科 Department of Medical Oncology, Beijing Chest Hospital, Capital Medical University, Beijing 101149, China; 4 101149 北京，首都医科大学附属北京胸科医院肿瘤研究中心 Cancer Research Center, Beijing Chest Hospital, Capital Medical University, Beijing 101149, China

**Keywords:** 肺肿瘤, 免疫治疗, 新辅助治疗, 手术, Lung neoplasms, Immunotherapy, Neoadjuvant therapy, Surgery

## Abstract

**背景与目的:**

初步研究证实新辅助免疫联合化疗对可手术非小细胞肺癌近期疗效显著，但国内相关临床试验较少。本研究回顾性分析应用新辅助免疫治疗联合化疗的可手术Ib期-IIIb期非小细胞肺癌的临床病理资料，初步评估新辅助免疫治疗联合化疗的疗效及安全性。

**方法:**

回顾性分析2019年11月-2020年12月期间于首都医科大学附属北京胸科医院胸外科治疗的临床分期Ib期-IIIb期的非小细胞肺癌患者20例，术前应用免疫联合化疗新辅助治疗，根据影像学和病理学方法分别评估疗效。

**结果:**

全组患者新辅助治疗后影像学评估疗效，客观有效率（objective response rate, ORR）为85.0%（完全缓解4例，部分缓解13例），疾病稳定1例（5.0%），疾病进展2例（10.0%）。其中17例后续接受手术治疗，16例达到R0（no residual tumor）切除，1例R1（microscopic residual tumor）切除。术后病理评估：主要病理缓解率（major pathologic response, MPR）为47.1%（8/17），其中完全病理缓解率（complete pathologic response, CPR）为29.4%（5/17）。主要不良反应：免疫相关性肺炎（Ⅳ级）1例，Ⅲ级及以上血液学毒性9例（45.0%）。

**结论:**

新辅助免疫联合化疗对于可手术的非小细胞肺癌近期疗效显著，具有一定的安全性及有效性。但新辅助免疫联合化疗的远期疗效、最佳周期数以及理想预测免疫治疗效果的标记物仍有待研究。

## Abstract (en)

**Background and objective:**

Preliminary researches conformed that neoadjuvant immunotherapy combined with chemotherapy had a significant short-term effect in resectable non-small cell lung cancer (NSCLC), but there were few clinical trials about neoadjuvant chemoimmunotherapy in China. We aimed to assess retrospectively the antitumour activity and safety of neoadjuvant chemoimmunotherapy for resectable stage Ib-IIIb NSCLC.

**Methods:**

Twenty patients who had been diagnosed as stage Ib-IIIb NSCLC and received chemoimmunotherapy as neoadjuvant treatment between November 2019 and December 2020, in Beijing Chest Hospital, Capital Medical University were recruited. These patients received neoadjuvant treatment for 21 days as a cycle and antitumour activity and safety were evaluated every two cycles.

**Results:**

Of 20 patients received neoadjuvant chemoimmunotherapy, 17 patients underwent surgical resection. 16 patients had R0 resection (no residual tumor resection) and 1 patient had R1 resection (microscopic residual tumor resection). Radiographic objective response rate (ORR) was 85.0% (4 complete response, 13 partial response). 5.0% (1/20) of patients had stable disease, and 10.0% (2/20) of patients had progression disease. The major pathologic response (MPR) was 47.1% (8/17), and complete pathologic response (CPR) was 29.4% (5/17). 1 case developed grade IV immune-related pneumonia (IRP) and 9 (45.0%) cases had grade III hematologic toxicity.

**Conclusion:**

Immunotherapy combined with chemotherapy as neoadjuvant therapy has a better efficiency and tolerable adverse effects for patients with resectable NSCLC in stage Ib-IIIb.

目前免疫检查点抑制剂已经成为晚期非小细胞肺癌（non-small cell lung cancer, NSCLC）的重要治疗方法，美国国立综合癌症网络（National Comprehensive Cancer Network, NCCN）指南对于晚期程序性死亡受体-配体1（programmed cell death ligand 1, PD-L1）阳性表达（≥1%）且表皮生长因子受体（epidermal growth factor receptor, *EGFR*）、间变性淋巴瘤激酶（anaplastic lymphoma kinase, *ALK*）、*c-ros*原癌基因1酪氨酸激酶（*c-ros* oncogene 1 receptor kinase, *ROS1*）、*BRAF*等基因表达阴性或未知的肺鳞癌，一线方案推荐首选卡铂+紫杉醇/白蛋白紫杉醇+帕博丽珠单抗治疗^[1]^。免疫治疗在不可手术切除肺癌中取得的良好疗效鼓舞研究者们开始探索其在可手术切除NSCLC中的作用。Forde等^[2]^首次提出免疫药物在NSCLC术前新辅助治疗中的有效性。随后，单药免疫、双联免疫和免疫联合化疗新辅助治疗等方案相继被提出，并已开展相关临床试验。初步研究^[3]^发现新辅助免疫治疗相较新辅助化疗有效性更高，尤其是免疫联合化疗方案更具有优势。但目前国内相关临床研究较少，刘雨桃等^[4]^对Ib期-IIIb期NSCLC采用程序性死亡受体1（programmed cell death 1, PD-1）单抗联合化疗方案给与新辅助治疗，结果显示具有疗效好、不良反应发生率低、主要病理缓解率（major pathologic response, MPR）高等特点。本研究中，我们回顾性分析首都医科大学附属北京胸科医院应用新辅助化疗联合免疫治疗的Ib期-IIIb期且病变可切除的NSCLC 20例，初步探讨PD-1单抗联合化疗作为Ib期-IIIb期NSCLC新辅助治疗方案的疗效和安全性。

## 1 资料与方法

### 1.1 纳入标准

回顾性分析2019年11月-2020年12月期间于北京胸科医院确诊的NSCLC、病变可切除并接受新辅助免疫治疗联合化疗的患者。纳入标准：①确诊为NSCLC，分期为Ib期-IIIb期；②术前经3名胸外科副高级别以上医师评估，认定病变可切除，但肿瘤体积 > 3 cm，中央型肺癌累及隆突、支气管或肺血管、肺门或纵隔淋巴结转移等；③卡氏体能状态评分（Karnofsky performance status, KPS）≥80分，可耐受新辅助治疗；④肝肾功能基本正常。排除标准：①具有远处转移或存在手术禁忌证的患者；②既往有恶性肿瘤病史；③患有自身免疫性疾病，或长期应用免疫抑制类药物的患者；④PD-（L）1单抗或化疗不耐受。

### 1.2 治疗方案

均采用免疫治疗联合含铂双药化疗。免疫药物均为PD-1抑制剂，包括帕博丽珠单抗、特瑞普利单抗、信迪利单抗和卡瑞利珠单抗。化疗方案参考晚期NSCLC一线方案，腺癌选择培美曲塞（500 mg/m^2^, d1）+顺铂（75 mg/m^2^, d1）/奈达铂（75 mg/m^2^, d1）；鳞癌多数选择白蛋白结合紫杉醇（260 mg/m^2^, d1）/紫杉醇脂质体（175 mg/m^2^, d1）+奈达铂（75 mg/m^2^, d1）或卡铂[曲线下面积（area under the curve, AUC）=5，d1]或洛铂（30 mg/m^2^, d1）。1例选择吉西他滨1, 000 mg/m^2^，d1，d8+奈达铂（75 mg/m^2^, d1）。免疫治疗和化疗均为21天为1个周期，每2个周期进行肿瘤疗效评估。

### 1.3 PD-L1检测

采用免疫组织化学染色法对肿瘤细胞进行PD-L1蛋白表达检测，以染色细胞百分比表示PD-L1蛋白的表达程度，染色细胞百分比 < 1%定义为阴性表达，染色细胞百分比≥1%定义为阳性表达（图 1）。

**图 1:**
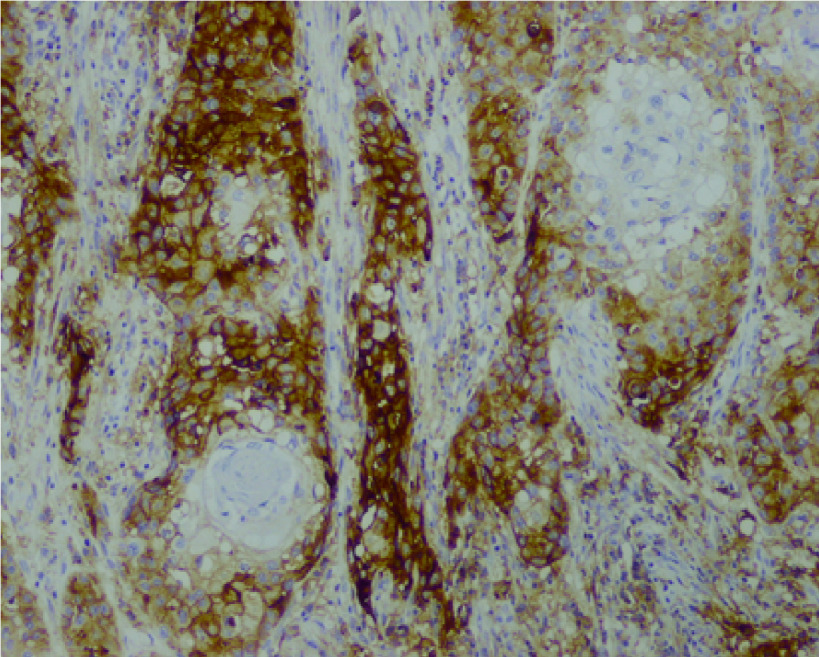
肿瘤细胞PD-L1表达（22C3染色，×10） PD-L1 expression in tumor cells (22C3 staining, ×10)

### 1.4 评估方法

每2个周期复查胸部计算机断层扫描（computed tomography, CT），按照实体瘤的评价标准1.1版（Response Evaluation Criteria in Solid Tumour 1.1, RECIST 1.1）进行疗效评估^[5]^。指标包括完全缓解（complete response, CR）、部分缓解（partial response, PR）、疾病稳定（stable disease, SD）、疾病进展（progressive disease, PD）、客观有效率（objective response rate, ORR）、疾病控制率（disease control rate, DCR）。术后病理评估指标：MPR和完全病理缓解率（complete pathological response, CPR）。MPR定义为新辅助治疗诱导的肿瘤消退在病理上残留肿瘤≤10%，CPR定义为新辅助治疗诱导的肿瘤消退在病理上未见残留肿瘤^[6, 7]^。

新辅助治疗结束到手术的时间（time to surgery, TTS）定义为从给药结束至接受外科手术的时间。自患者使用本研究所观察药物开始，至治疗结束后1个月内，发生的任何不良反应，无论与试验药物是否有因果关系，均判定为不良事件。不良事件评价按美国国立癌症研究所通用毒性标准3.0版本标准进行。

### 1.5 统计学方法

采用SPSS 23.0统计软件对数据进行分析。疗效比较采用*χ*^2^检验或*Fisher*检验，检验水准为α=0.05。

## 2 结果

### 2.1 全组患者的基线特征

根据纳入与排除标准，应用术前新辅助免疫治疗患者共20例，患者详细信息如表 1。其中男性17例，女性3例；≥60岁12例， < 60岁8例。鳞癌17例，腺癌2例，因病理组织过少，未确定病理类型1例。接受1个周期治疗2例，2个周期治疗3例，3个周期治疗10例，4个周期治疗5例。PD-L1检测18例，其中阳性11例，阴性7例。

**表 1:** 20例患者的临床资料 Clinical characteristics of the 20 patients

Characteristics	All patients (*n*=20)
Age (yr)	
< 60	8 (40.0%)
≥60	12 (60.0%)
Gender	
Male	17 (85.0%)
Female	3 (15.0%)
Histologic type	
Squamous cell carcinoma	17 (85.0%)
Adenocarcinoma	2 (10.0%)
Others	1 (5.0%)
Clinical disease stage	
Ib	4 (20.0%)
IIIa	12 (60.0%)
IIIb	4 (20.0%)
Smoking status	
Never	3 (15.0%)
Former or current	17 (85.0%)
PD-L1	
Positive	11 (55.0%)
Negative	7 (35.0%)
No data	2 (10.0%)
Cycles of neoadjuvant therapy	
1+2	5 (25.0%)
3	10 (50.0%)
4	5 (25.0%)
PD-L1: programmed cell death ligand 1.	

### 2.2 新辅助治疗疗效评估

根据RECIST 1.1评估，CR 4例（20.0%），PR 13例（65.0%）（图 2），SD 1例（5.0%），PD 2例（10.0%）。ORR为85.0%，DCR为90.0%。2例患者新辅助治疗后肿瘤进展，1例原发灶加重，另1例原发灶明显缩小，但对侧纵隔淋巴结出现新发转移。

**图 2:**
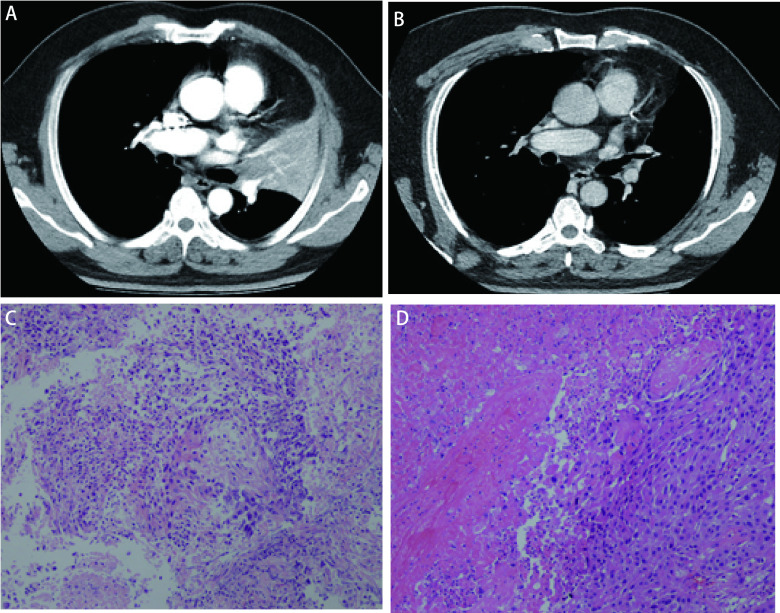
1例患者新辅助治疗后影像评估及病理评估。A：治疗前，CT扫描显示左肺上叶中心型肿物，阻塞性肺不张；B：新辅助治疗12周（手术前），新辅助治疗后左肺上叶肿物明显缩小，上叶支气管通畅，管壁粗糙；C：治疗前支气管镜活检病理鳞癌（HE染色，×10）；D：手术切除术后病理评估残存肿瘤细胞5%（HE染色，×10）。 Patterns of radiologic and pathological response to neoadjuvant therapy from 1 patient. A: (Pretreatment imaging) CT scan performed before treatment shows a primary tumor mass and obstructive atelectasis in the upper left lobe of the lung; B: (Week 12 after neoadjuvant therapy) CT scan showed a significant shrinkage of primary tumor mass before surgery; C: (Pretreatment tumor biopsy) squamous-cell carcinoma conformed by biopsy in bronchoscopy before treatment (HE staining, ×10); D: (Resection specimen) in the post-treatment specimen, there was 5% residual tumor cells (HE staining, ×10). CT: computed tomography.

### 2.3 手术治疗

全组17例患者接受手术，手术切除率为85%，末次治疗到手术的中位时间为38 d（21 d-53 d）。手术方式：单纯肺叶切除10例，袖式肺叶切除1例，复合肺叶切除3例，左全肺切除4例，其中2例为心包内左全肺切除术。肺叶切除合并肺动脉成形3例，合并奇静脉切除3例。R0（no residual tumor）切除16例，R1（microscopic residual tumor）切除1例。无围术期死亡。无呼吸衰竭、肺炎及肺不张等并发症。术后均定期随访，无复发及转移。

### 2.4 病理评估

MPR 8例（47.1%）（图 2），其中CPR 5例（29.4%），未达MPR 9例（52.9%）。

### 2.5 不良反应

本研究不良反应多与化疗药物相关，且多为Ⅰ级-Ⅱ级，Ⅲ级及以上不良反应发生率相对较低，包括9例白细胞减少、中性粒细胞减少，血小板降低。严重免疫相关不良反应1例，为Ⅳ级免疫相关性肺炎，停用免疫药物，应用甲泼尼龙琥珀酸钠治疗，1周后病情好转，后期改用新辅助化疗。甲状腺功能低下1例。

## 3 讨论

目前免疫检查点抑制剂在不可切除肺癌中取得良好疗效^[1]^，激励学者们探索其在可切除NSCLC中的作用。研究^[8]^显示，手术前肿瘤内存在多数表达免疫检查点抑制剂靶标的细胞，进而在免疫治疗时大量的肿瘤抗原有助于激活大量肿瘤浸润淋巴细胞，引发持久的抗肿瘤效应。术前诱导的系统性免疫反应可使机体产生长期免疫记忆，预防肿瘤复发，而术后患者因肿瘤的切除无法产生免疫介导的持续的抗肿瘤效应。因此，新辅助免疫治疗有一定合理性。目前多项II期临床研究^[9, 10]^也提示新辅助免疫治疗具有显著的近期疗效。

目前肺癌新辅助免疫治疗主要有三种模式：单免疫药治疗、双联免疫药治疗和免疫联合化疗^[11]^。在Check Mate159研究^[2]^，应用纳武单抗单药免疫新辅助治疗，MPR达到45%（9/20）。NEOSTAR研究^[12]^对比双联免疫与单药免疫新辅助的疗效，在接受手术的39例患者中，双药纳武单抗+伊匹木单抗治疗的MPR与CPR优于单药纳武单抗组（44% *vs* 19%）。而NADIM研究^[13]^新辅助免疫联合化疗在IIIa期NSCLC的疗效，与前二种模式相比较，免疫联合化疗效果更优，MPR为83%，CPR为71%。且药物毒性反应较小。本组病例RECIST评估ORR为85.0%，DCR为90.0%。手术后病理评估MPR为47.1%（8/17），其中CPR为29.4%（5/17）。低于NADIM研究的结果，这可能与本研究的样本量较小有关，但仍明显高于术前新辅助化疗的MPR^[14]^。

新辅助治疗的主要目的之一是使肿瘤缩小、分期降低，提高手术的可切除性^[15]^。因此，ORR也是新辅助免疫治疗重要的评估指标之一。目前，肺癌新辅助免疫治疗不同模式间ORR存在明显差异。单纯免疫药物ORR仅为10%^[2]^，双免疫药物联合应用ORR为19%^[12]^。而采用免疫药物联合含铂双药的方案ORR可达到78%^[13]^。本研究ORR为85%，其中CR 4例（20.0%）。17例手术中16例达到R0切除，有4例IIIa期及2例IIIb期患者获得MPR，手术切除性提高。而且，免疫药物联合含铂双药的新辅助治疗方案，获得较高MPR可能转化为生存获益^[16]^。因此，免疫药物联合化疗方案可能是新辅助免疫治疗更合理的选择。

NCCN指南建议新辅助化疗为4个周期，而新辅助免疫治疗的疗程尚无定论，目前多数研究选择2个周期-4个周期。周期太短，免疫治疗可能尚未发挥作用；周期太长，肿瘤可能进展从而失去手术机会。本研究中，15例患者接受3个周期-4个周期新辅助治疗，其中10例在2个周期治疗后病变不再缩小，4例在3个周期不再缩小。而且，1例患者治疗4个周期后病变进展，失去手术机会。另有3例患者治疗3个或4个周期后病变增大，但未达到进展标准。延长周期数是否提高MPR也有待进一步证实。CheckMate-159试验^[2]^以及LCMC3^[17]^试验，均给予2个周期治疗，MPR分别为45%和19%，而NADIM研究^[13]^给予3个周期治疗，MPR为83%。本研究中，3例2个周期治疗患者中1例MPR，10例3个周期化疗患者6例MPR，其中4例CPR。5例4个周期治疗患者中4例接受手术，只有1例CPR。疗程增加并没有提高MPR。因此，新辅助免疫治疗的合理疗程尚需大样本随机对照研究证实。

PD-L1的表达能否预测免疫治疗的效果尚存在争议。本研究中共有18例患者检测PD-L1表达情况，经RECIST评估后，11例表达阳性患者ORR为100%（9例PR，2例CR）。7例表达阴性患者ORR为57.1%（3例PR，1例CR）。PD-L1表达与影像学评估疗效相关，差异有统计学意义（*P*=0.043）。而刘雨桃等^[4]^研究未显示PD-L1≥1%与PD-L1 < 1%以及PD-L1≥10%与PD-L1 < 10%患者ORR及DCR间差异有统计学意义。PD-L1阳性组9例接受手术，病理评估5例为MPR，其中CPR 2例。PD-L1阴性组6例接受手术，仍有1例达到CPR。PD-L1表达与病理学疗效无明显相关（*P*=0.580）。表明PD-L1表达并不是免疫治疗是否有效的良好预测指标，即使表达阴性也不能否定免疫联合化疗新辅助的治疗效果。在CheckMate 159研究^[2]^及LCMC3研究^[17]^中也未观察到PD-L1表达与MPR的相关关系，而NEOSTAR研究^[12]^发现获得MPR的患者治疗前PD-L1表达较高，且在PD-L1 > 1%的患者中治疗后肿瘤残余更少。因此，还需要寻找其他预测免疫治疗效果的标记物。

免疫治疗的不良反应也是值得关注的问题，严重的治疗毒性反应可能会导致手术延期，甚至错失手术机会^[18]^。NADIM研究^[13]^中接受免疫治疗联合化疗的患者均及时接受了手术，未因疾病进展或毒性反应而提前退出研究。而NEOSTAR研究^[12]^，治疗相关副反应导致22%的患者发生手术延期，延期时间长达42 d。而本研究有1例患者出现Ⅳ级免疫相关性肺炎，被迫中止免疫治疗。2例患者治疗后疾病进展，1例失去手术机会。因此，新辅助免疫治疗可能导致手术延迟或失去手术机会，选择入组人群应慎重考虑。

综上所述，本研究虽为小样本临床研究，初步结果显示，新辅助免疫联合化疗对于可手术的NSCLC近期疗效显著，具有一定的安全性及有效性。但新辅助免疫联合化疗在可手术的NSCLC的远期疗效、最佳周期数以及预测免疫治疗效果的标记物仍需进一步研究证实。
